# Progression of the ascending aorta diameter after surgical or transcatheter bicuspid aortic valve replacement

**DOI:** 10.1093/icvts/ivae100

**Published:** 2024-05-22

**Authors:** Giovanni Alfonso Chiariello, Michele Di Mauro, Annalisa Pasquini, Piergiorgio Bruno, Marialisa Nesta, Ludovica Fabiani, Andrea Mazza, Martina Meloni, Elisabetta Baldo, Myriana Ponzo, Francesco Ferraro, Antonio Davide Conserva, Edoardo D’Acierno, Emmanuel Villa, Carlo Trani, Francesco Burzotta, Massimo Massetti

**Affiliations:** Department of Cardiovascular Sciences, Agostino Gemelli Foundation Polyclinic IRCCS, Rome, Italy; Catholic University of the Sacred Heart, Rome, Italy; Heart and Vascular Centre, Cardiovascular Research Institute, CARIM, Maastricht, Netherlands; Department of Cardiovascular Sciences, Agostino Gemelli Foundation Polyclinic IRCCS, Rome, Italy; Catholic University of the Sacred Heart, Rome, Italy; Department of Cardiovascular Sciences, Agostino Gemelli Foundation Polyclinic IRCCS, Rome, Italy; Catholic University of the Sacred Heart, Rome, Italy; Department of Cardiovascular Sciences, Agostino Gemelli Foundation Polyclinic IRCCS, Rome, Italy; Catholic University of the Sacred Heart, Rome, Italy; Department of Cardiovascular Sciences, Agostino Gemelli Foundation Polyclinic IRCCS, Rome, Italy; Catholic University of the Sacred Heart, Rome, Italy; Department of Cardiovascular Sciences, Agostino Gemelli Foundation Polyclinic IRCCS, Rome, Italy; Catholic University of the Sacred Heart, Rome, Italy; Department of Cardiology, Thoracic and Vascular Sciences, S. Matteo University Hospital, Pavia, Italy; Department of Cardiology, Cristo Re Hospital, Rome, Italy; Department of Cardiology, Cristo Re Hospital, Rome, Italy; Department of Cardiovascular Sciences, Agostino Gemelli Foundation Polyclinic IRCCS, Rome, Italy; Catholic University of the Sacred Heart, Rome, Italy; Department of Cardiovascular Sciences, Agostino Gemelli Foundation Polyclinic IRCCS, Rome, Italy; Catholic University of the Sacred Heart, Rome, Italy; Department of Cardiovascular Sciences, Agostino Gemelli Foundation Polyclinic IRCCS, Rome, Italy; Catholic University of the Sacred Heart, Rome, Italy; Department of Cardiovascular Surgery, Cardiac Surgery Unit, Poliambulanza Foundation Hospital, Brescia, Italy; Department of Cardiovascular Sciences, Agostino Gemelli Foundation Polyclinic IRCCS, Rome, Italy; Catholic University of the Sacred Heart, Rome, Italy; Department of Cardiovascular Sciences, Agostino Gemelli Foundation Polyclinic IRCCS, Rome, Italy; Catholic University of the Sacred Heart, Rome, Italy; Department of Cardiovascular Sciences, Agostino Gemelli Foundation Polyclinic IRCCS, Rome, Italy; Catholic University of the Sacred Heart, Rome, Italy

**Keywords:** Bicuspid aortic valve, Aortic stenosis, Aortic dilatation, Aortic valve replacement, Transcatheter aortic valve implantation

## Abstract

**OBJECTIVES:**

Ascending aorta (AA) dilatation in patients with bicuspid aortic valve (AV) is related both to genetic and haemodynamic factors. The aim of this study is to compare late progression of AA dilatation in bicuspid AV patients undergoing surgical aortic valve replacement (SAVR) versus transcatheter aortic valve implantation (TAVI).

**METHODS:**

Data of 189 consecutive patients who underwent AV replacement for severe bicuspid AV stenosis were prospectively collected. Patients who underwent SAVR were compared to patients who underwent TAVI. Indication to the procedure was validated by the institutional Heart Team. Aortic diameters were evaluated by transthoracic echocardiogram. Differences between preoperative and long-term follow-up AA diameters were compared in the 2 groups.

**RESULTS:**

Between January 2015 and December 2021, 143 (76%) patients underwent SAVR and 46 (24%) patients underwent TAVI. At 4.6 (standard deviation 1.7) years follow-up, patients in the TAVI group showed significantly lower survival (*P* = 0.00013) and event-free survival (*P* < 0.0001). AA diameter progression was lower in surgical compared to transcatheter patients, 0.95 (0.60, 1.30) vs 1.65 (0.67, 2.63) mm, *P* = 0.02. AA diameter progression indexed for body surface area and height was lower in the surgical group: 0.72 (0.38, 1.05) vs 1.05 (0.39, 1.71) mm/m^2^, *P* = 0.02, and 0.59 (0.36, 0.81) vs 1.11 (0.44, 1.78) mm/m, *P* = 0.001, respectively. At multivariable linear regression analysis transcatheter procedure, baseline aortic diameter and paravalvular leak were significantly associated with increased postoperative AA dilatation.

**CONCLUSIONS:**

Bicuspid AV patients who underwent SAVR, showed significantly less long-term AA diameter progression than patients who underwent transcatheter procedure.

## Graphical Abstract

**Figure:**
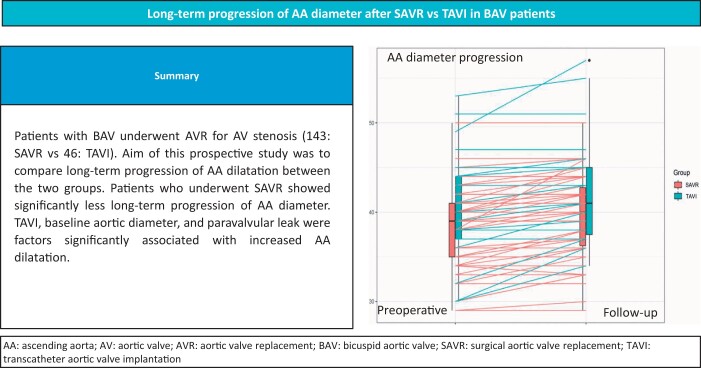

## INTRODUCTION

Bicuspid aortic valve (BAV) is the most common congenital valve defect, with a prevalence of 1 and 2% [1, 2]. It may cause aortic valve (AV) stenosis, AV regurgitation and in 20–84% of cases is associated with dilatation of the ascending aorta (AA), named bicuspid aortopathy [3–5]. Most frequently, dilatation of the AA is limited to its tubular tract (70% of cases) and its risk increases progressively with age and if concomitant AV stenosis is associated [3–7]. Bicuspid aortopathy can be related to genetic molecular factors, histologic changes of the weakened aortic media and to the haemodynamic characteristics, that is, the turbulent flow and asymmetric shear stress on the aortic wall caused by the AV-altered morphology [3–5, 7, 8]. In most operable BAV patients with indication to aortic valve replacement (AVR) for severe AV stenosis, so far surgical aortic valve replacement (SAVR) represented the gold standard treatment. However, in recent years, as an alternative to surgery, transcatheter aortic valve implantation (TAVI) has been proposed also in patients with BAV [6, 8–11].

In patients with BAV and AV stenosis undergoing surgical or percutaneous treatment, possibility and severity of subsequent dilatation of the AA is matter of debate and data are still scarce. Recent studies on the dilatation of the AA post-AVR are controversial, especially in comparing patients with BAV and patients with tricuspid AV [12–19]. At the moment a comparison between SAVR versus TAVI in BAV patients focused on the difference in aortic diameter late progression is not available.

In this prospective study, in patients with BAV who underwent either SAVR or TAVI, we intend to compare along with the long-term clinical outcome and haemodynamic results of treatment, the late dilatation of the AA in the 2 groups of patients.

## MATERIALS AND METHODS

### Ethics statement

The study was designed in accordance to the Declaration of Helsinki and Good Clinical Practice Guidelines. The Institutional Review Board and Ethics Committee of the Center approved the study protocol (ID 5239, Number protocol 0038232/22, ClinicalTrials.gov Identifier: NCT05708118). The patients provided written informed consent for surgery and follow-up investigations and to use their data for scientific purposes. Patients signed a written informed consent form at follow-up, to be involved in this study.

### Methods

This is a prospective observational, non-randomized single-centre study. Between January 2015 and December 2021, 189 patients with congenital BAV underwent isolated AVR, either with SAVR or with TAVI. The treatment indication was validated by a dedicated Institutional Heart Team. Valve prosthesis selection was performed according to the 2021 *European Journal of Cardio-thoracic Surgery* expert consensus statement [20]. In surgical AVR, the operation was performed through median sternotomy, with cardiopulmonary bypass and crystalloid cardioplegia. In selected patients a mini-sternotomy approach with central arterial cannulation and central or femoral cannulation for venous drainage was used. Either a stented bioprosthesis or a mechanical prosthesis was implanted in supra-annular position. Patients selected for TAVI underwent a transfemoral, transapical, or trans-carotid approach and either a self-expandable or a balloon-expandable prosthesis was implanted following the manufacturer's best practice recommendations (Supplementary Material, Table S1). Combined procedures, reinterventions, patients with endocarditis, aortic dissections, Marfan syndrome or other connective tissue disorders were excluded from the study.

In SAVR patients who underwent bioprosthetic AVR, subcutaneous enoxaparin was administered early after intervention, subsequently replaced by warfarin for 3 months and by life-long acetyl-salicylic acid at 100 mg/day. In patients who underwent mechanical AVR, life-long warfarin therapy was administered. TAVI patients were treated with life-long acetyl-salicylic acid at 100 mg/day. As regards antihypertensive therapy, it is generally tailored to the patient's clinical profile. However, our approach entails the initial administration of an ace-inhibitor or a sartan, and a beta-blocker. Subsequently, in the second instance, we consider the administration of calcium channel blockers and alpha-blockers.

Baseline preoperative data included age, sex, body mass index, body surface area (BSA), cardiovascular risk factors, comorbid conditions, New York Heart Association (NYHA) functional class, Euroscore II, preoperative echocardiographic data. Aortic diameters were evaluated by transthoracic echocardiogram, performed by a core-lab of selected and experienced cardiologists dedicated to echocardiographic evaluation of cardiac surgery patients. The tubular AA diameter was measured from the parasternal long axis view, at end-diastolic phase, ∼1 cm above the sinotubular junction, using the leading edge-to-leading edge criteria. In each measurement the maximum diameter of the AA was considered.

Transprosthetic gradients were calculated at echocardiogram using the modified Bernoulli equation. Effective orifice area (EOA) was calculated using the continuity equation. Severe prosthesis–patient mismatch was defined as EOA indexed by BSA < 0.65 cm^2^/m^2^. Hematological and biochemical investigations, 12-lead electrocardiography and chest radiography were performed. In addition to preoperative coronary angiography, computed tomography was performed in all TAVI patients and, when required, in SAVR patients. Early postoperative data and significant perioperative complications were recorded. Transthoracic echocardiography was performed preoperatively, early in the postoperative period, at discharge and at follow-up. All AA diameters were measured by echocardiography. Although computed tomography scan and magnetic resonance imaging may provide excellent accuracy of aortic diameter measurements, the more practical transthoracic echocardiography is considered in literature a very reliable tool for AA diameters evaluation [21, 22].

Clinical and echocardiographic follow-up extended for 4.6 (standard deviation 1.7 years). Events considered were mortality, cardiovascular mortality, incidence of pacemaker implantation, neurological events, myocardial infarction, paravalvular regurgitation, reintervention for bioprosthetic dysfunction. The clinical follow-up was 100% complete, the echocardiographic examination was obtained in 178 (94%) patients. Survival, clinical status, NYHA functional class, late complications, reinterventions in the 2 groups were compared. Echocardiographic parameters included haemodynamic prosthetic data (EOA; transprosthetic peak pressure gradient and transprosthetic mean pressure gradient), biventricular function, AA diameters, ascending aorta diameter indexed to height (AA/h), and ascending aorta diameter indexed to body surface area (AA/BSA). Aortic diameter progression was defined as the difference between follow-up and preoperative diameters.

### Statistical analysis

Continuous variables are expressed as median and quartiles, for normally and were compared using the Mann–Whitney *U* test. Proportions are expressed as percentages and compared using χ+ test or Fisher exact test, as appropriate. Changes in AA dimensions over time were modelled using mixed linear model analyses with a subject as random effect. Independent variables investigated were age, female gender, hypertension, TAVI (versus SAVR), baseline AA dimension, baseline mean pressure gradient, severe aortic regurgitation, paravalvular leak and time. The final models were realized by stepwise elimination using a threshold *P*-value of <0.10. Paired comparison was performed with paired t-test or Wilcoxon signed-rank test according to the distribution of evaluated parameters. Survival and event-free survival were plotted using Kaplan–Meier curves with log-rank test. R-studio version 1.1.463 (2009–2018) was used for all statistical analyses. The significance of differences was considered at a *P*-value of <0.05.

## RESULTS

### Patients

In the study period, 189 patients with congenital BAV underwent isolated AVR. Out of this sample, 143 (76%) patients underwent SAVR and 46 (24%) underwent TAVR. Clinical characteristics of the 189 patients with BAV who underwent AVR are summarized in Table 1. Median age was 66 (59, 72) years for SAVR patients vs 78 (73, 83) years for TAVI patients, *P* ≤ 0.001. In the SAVR group, 97 (68%) patients were male, and in the TAVI group, 28 (61%) were male (*P* = 0.47).

**Table 1:** Patients characteristics

	SAVR, *N* = 143	TAVI, *N* = 46	*P*-value
Age (years), median (IQR)	66 (59, 72)	78 (73, 83)	<0.001
Male gender, *n* (%)	97 (68)	28 (61)	0.47
Height (cm), median (IQR)	170 (164, 174)	165 (160, 170)	0.002
BMI (kg/m^2^), median (IQR)	26.0 (24.1, 28.4)	26.7 (23.4, 30.3)	0.72
BMI > 30 kg/m^2^, *n* (%)	19 (13)	12 (26)	0.044
BSA (cm^2^), median (IQR)	1.86 (1.72, 1.98)	1.78 (1.64, 1.92)	0.045
Hypertension, *n* (%)	107 (75)	40 (87)	0.085
Diabetes, *n* (%)	23 (16)	8 (17)	0.83
Dislipidaemia, *n* (%)	69 (48)	25 (54)	0.58
Smoke, *n* (%)	77 (54)	24 (52)	0.84
History of CAD, *n* (%)	25 (17)	13 (28)	0.11
Previous neurologic events, *n* (%)	13 (9)	3 (6)	0.81
PVD, *n* (%)	3 (2)	10 (22)	<0.001
Creatinine (mg/dl), median (IQR)	0.89 (0.7, 1)	1.07 (0.92, 1.36)	<0.001
EGFR < 30, *n* (%)	6 (4.2)	11 (24)	<0.001
COPD, *n* (%)	16 (11)	8 (17)	0.33
NYHA class, *n* (%)			<0.001
I	35 (24)	1 (2.2)	
II	64 (45)	17 (37)	
III	39 (27)	26 (57)	
IV	5 (3)	2 (4)	
Angina, *n* (%)	26 (18)	6 (13)	0.43
Syncope, *n* (%)	22 (15)	10 (22)	0.31
Euroscore II (%), median (IQR)	1.15 (0.8, 1.7)	3.78 (1.9, 6)	< 0.001
Sinus rhythm, *n* (%)	133 (93)	30 (65)	<0.001
Rhythm other than sinus, *n* (%)	44 (31)	36 (78)	<0.001
Aortic stenosis regurgitation, *n* (%)	100 (70)	33 (72)	0.81
AV MPG (mmHg), median (IQR)	57 (48, 66)	49 (41, 59)	0.012
AV PPG (mmHg), median (IQR)	94 (86, 110)	80 (65, 97)	<0.001
AVA (cm^2^), median (IQR)	0.7 (0.6, 0.8)	0.7 (0.6, 0.8)	0.064
BV Type 0, *n* (%)	29 (20)	0 (0)	<0.001
BV Type 1, *n* (%)	112 (78)	45 (98)	0.02
BV Type 2, *n* (%)	2 (1)	1 (2)	0.13
LVEF (%), median (IQR)	65 (60, 70)	58 (40, 64)	<0.001
LVEDD (mm), median (IQR)	51 (46, 56)	49 (45, 55)	0.82
LVESD (mm), median (IQR)	29 (25, 35)	33 (29, 40)	0.012
IVS (mm), median (IQR)	14 (13, 16)	14 (13, 15)	0.60
LVPWD (mm), median (IQR)	13 (12, 14)	12 (11, 13)	0.31
LV mass (g), median (IQR)	265 (218, 321)	259 (209, 304)	0.42
AV annulus (mm), median (IQR)	23 (22, 25)	22 (21, 24)	0.036
AA diameter (mm), median (IQR)	39 (35, 42)	40 (37, 43.8)	0.040
AA/BSA (mm/m^2^), median (IQR)	20.9 (19, 22.7)	21.8 (20.6, 24)	0.006
AA/h (mm/cm), median (IQR)	22.7 (20.8, 24.6)	23.9 (22.8, 26.8)	0.003
STJ diameter (mm), median (IQR)	29 (26, 31)	34 (28, 41)	0.074
AR diameter (mm), median (IQR)	36 (33, 38)	37 (33, 40)	0.30

AA: ascending aorta; AA/BSA: ascending aorta diameter indexed to body surface area; AA/h: ascending aorta diameter indexed to height; AR: aortic root; AV: aortic valve; AV MPG: aortic valve mean pressure gradient; AV PPG: aortic valve peak pressure gradient; AVA: aortic valve area; BMI: body mass index; BSA: body surface area; BV: bicuspid valve; CAD: coronary artery disease; COPD: chronic obstructive pulmonary disease; EGFR: estimated glomerular filtration rate; IQR: interquartile range; IVS: interventricular septum; LV: left ventricle; LVEDD: left ventricular end-diastolic diameter; LVEF: left ventricular ejection fraction; LVESD: left ventricular end-systolic diameter; LVPWD: left ventricular posterior wall diameter; NYHA: New York Heart Association functional class; PVD: peripheral vascular disease; SAVR: surgical aortic valve replacement; STJ: sinotubular junction; TAVI: transcatheter aortic valve implantation.

No significantly different incidence of hypertension and diabetes was observed. The TAVI patients were older and in worse clinical baseline conditions, with a significatively higher incidence of peripheral vascular disease and creatinine level, lower left ventricular ejection fraction and, as expected, presented a higher Euroscore II, 1.15 (0.80, 1.70) % in the SAVR group vs 3.78 (1.9, 6) % in the TAVI group, *P* ≤ 0.001.

According to Sievers classification [23], BAV type 0 was observed in 29 (20%) patients of the SAVR group vs 0 patients in the TAVI group (*P* ≤ 0.001). Type 1 BAV was observed in 112 (78%) SAVR patients vs 45 (98%) TAVI patients, *P* = 0.02, type 2 BAV in 2 (1%) SAVR patients vs 1 (2%) TAVI patients, *P* = 0.1.

The preoperative aortic root diameter was comparable in the 2 groups. TAVI patients presented a slight but significantly higher AA diameter, 39 (35, 42) mm in the SAVR group vs 40 (37, 43.8) mm in the TAVI group, *P* = 0.040. Preoperative AA/h was 22.7 (20.8, 24.6) mm/m in the SAVR group vs 23.9 (22.8, 26.8) mm/m in the TAVI group (*P* = 0.003); AA/BSA was 20.9 (19, 22.7) mm/m^2^ vs 21.8 (20.6, 24) mm/m^2^, *P* = 0.006.

### Operative data and 30-day outcome

In SAVR patients a bioprosthesis was implanted in 125 (87%) patients, a bileaflet mechanical prosthesis in 18 (13%). A stented bioprosthesis was used in 122 (85%) patients. One patient received a stentless bioprosthesis, and 2 a rapid-deployment prosthesis. Sizes 21 and 23 mm were most frequently used, having been selected for 38 (27%) and 66 (46%) patients, respectively. The mini-sternotomy approach was used in 75 (52%) patients. Cardiopulmonary bypass time was 108 (100, 122) min, and aortic cross-clamping time was 83 (70, 93) min.

In TAVI patients, a self-expandable prosthesis was implanted in 36 (78%) patients, and a balloon-expandable prosthesis in 10 (22%). Larger size valves were implanted, with 20 (44%) patients receiving a 29-mm prosthesis and 10 (22%) a 34-mm prosthesis. Forty (87%) patients underwent a transfemoral implantation; a transapical or trans-carotid approach was used in 5 (11%) and 1 (2%) patients, respectively.

The intensive care unit stay was significantly longer in SAVR patients, 2 (2, 3) vs TAVI 1.5 (1, 2) days, *P* ≤ 0.001. Surprisingly, in-hospital stay length was superior for TAVI versus SAVR patients, 12 (7, 18) vs 8 (7, 10) days, respectively, *P* = 0.001, and the cause is to be attributed to their age, fragility, and with greater comorbidities with a worst general clinical status. No significant difference was observed in 30-day mortality and incidence of 30-day cardiovascular events. One (1%) in-hospital death (*P* ≥ 0.9) and 1 (1%) perioperative myocardial infarction occurred in the SAVR group (*P* ≥ 0.9). In 2 (4%) TAVI patients a cerebral transient ischaemic attack was observed (*P* = 0.8). In the TAVI group, transprosthetic peak pressure gradient was 15 (12, 20) vs 23 (17, 32) mmHg, and in the SAVR group, *P* ≤ 0.001; transprosthetic mean pressure gradient was 8 (6, 11) vs 14 (10, 18) mmHg, *P* ≤ 0.001.

### Follow-up

Follow-up data with events occurred are reported in Table 2. Compared to TAVI patients, SAVR patients showed significantly better survival (*P* = 0.00013) and event-free survival (*P* ≤ 0.0001), (Supplementary Material, Figs S1 and S2). In the SAVR group, 3 (2%) patients died, vs 5 (11%) patients in the TAVI group. Cardiovascular mortality was comparable, 1 patient in the SAVR group and 2 patients in the TAVI group died for myocardial infarction. TAVI patients showed a significant higher incidence of permanent pacemaker implantation, 1 (1%) patient in the SAVR group vs 10 (22%) patients in the TAVI group. Incidence of neurovascular events was higher in the TAVI group, 1 (1%) patients in the SAVR group vs 4 (9%) patients in the TAVI group. Furthermore, TAVI patients presented a higher risk of mild or significant paravalvular regurgitation (paravalvular leak). Patients in the SAVR group showed a better clinical status with a significant improvement in symptoms (NYHA functional class).

**Table 2:** Follow-up results and events occurred

	SAVR, *N* = 139	TAVI, *N* = 41	*P*-value
Follow-up all-cause mortality, *n* (%)	3 (2)	5 (11)	0.020
Follow-up cardiovascular mortality, *n* (%)	1 (1)	2 (4)	0.067
Permanent pacemaker implantation, *n* (%)	1 (1)	10 (24)	<0.0001
Neurologic events, *n* (%)	1 (1)	4 (10)	0.019
Myocardial infarction, *n* (%)	1 (1)	0 (0)	>0.90
Paravalvular regurgitation, *n* (%)	5 (4)	15 (37)	<0.0001
Mild paravalvular regurgitation, *n* (%)	5 (4)	10 (24)	0.0002
Significant paravalvular regurgitation, *n* (%)	0 (0)	5 (12)	0.002
Reintervention for bioprosthetic valve dysfunction, *n* (%)	1 (1)	2 (5)	0.067
Aortic dissection, *n* (%)	0 (0)	0 (0)	–
NYHA functional class ≥III, *n* (%)	7 (5)	7 (17)	0.028
tMPG (mmHg), mean ± SD	14 ± 8	10 ± 4	0.015
EOA (cm^2^), mean ± SD	1.5 ± 0.3	1.7 ± 0.3	0.076
Severe PPM, *n* (%)	19 (14)	5 (12)	0.80

EOA: effective orifice area; NYHA: New York Heart Association; PPM: prosthesis–patient mismatch; tMPG: transprosthetic mean pressure gradient; SAVR: surgical aortic valve replacement; SD: standard deviation; TAVI: transcatheter aortic valve implantation.

**Figure 1:**
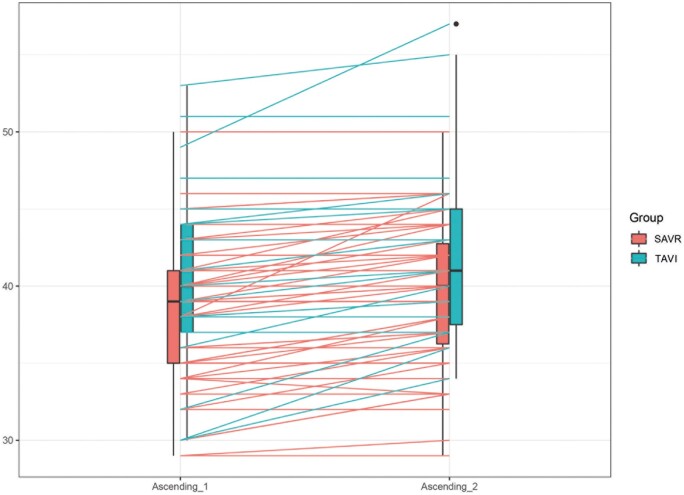
Box plot showing difference in progression of AA diameter in SAVR versus TAVI. Ascending_1: preoperative AA diameter; Ascending_2: follow-up AA diameter. AA: ascending aorta; SAVR: surgical aortic valve replacement; TAVI: transcatheter aortic valve implantation.

**Figure 2:**
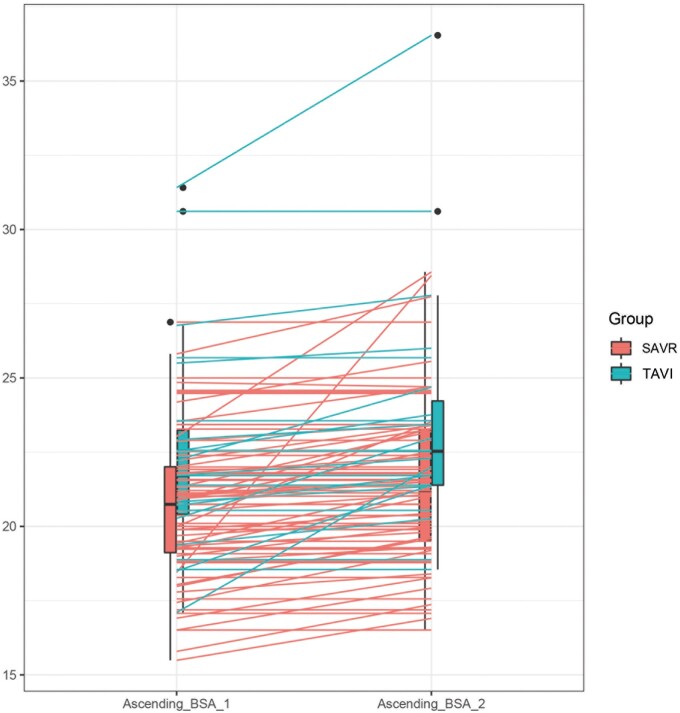
Box plot showing difference in progression of AA/BSA. in SAVR versus TAVI. Ascending_BSA_1: preoperative AA diameter indexed for BSA; Ascending_2_BSA: follow-up AA diameter indexed for BSA. AA: ascending aorta; AA/BSA: ascending aorta diameter indexed to body surface area; BSA: body surface area; SAVR: surgical aortic valve replacement; TAVI: transcatheter aortic valve implantation.

**Figure 3:**
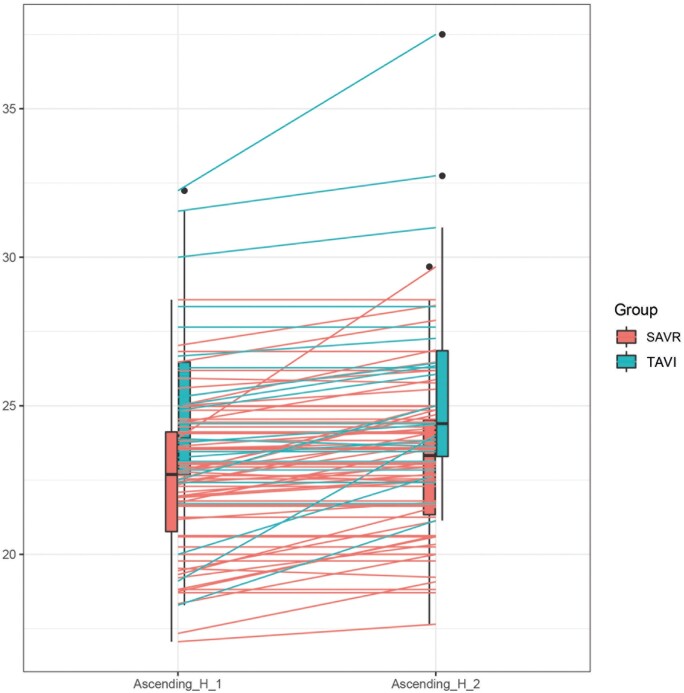
Box plot showing difference in progression of AA/h in SAVR versus TAVI. Ascending_H_1: preoperative AA diameter indexed for height; Ascending_H_2: follow-up AA diameter indexed for height. AA: ascending aorta; AA/h: ascending aorta diameter indexed to height; SAVR: surgical aortic valve replacement; TAVI: transcatheter aortic valve implantation.

At the echocardiographic follow-up, no significant difference was observed in aortic root progression, 0.87 (0.34, 1.39) mm in the SAVR group vs 1.35 (0.25, 2.44) mm in the TAVI group (*P* = 0.08). A significant progression of the AA diameter was observed in both groups. AA diameter increased from 39 (35, 42) to 39.9 (36, 43) mm in the SAVR group (*P* ≤ 0.001) and from 40 (37, 43.8) to 41.6 (37, 45) mm in the TAVI group (*P* = 0.002). However, compared to TAVI patients, SAVR patients showed lower AA diameter progression compared to TAVI patients: SAVR 0.95 (0.60, 1.30) vs TAVI 1.65 (0.67, 2.63) mm, *P* = 0.02. AA/BSA was 0.72 (0.38, 1.05) mm/m^2^ in the SAVR group vs 1.05 (0.39, 1.71) mm/m^2^ in the TAVI group (*P* = 0.02). AA/h was 0.59 (0.36, 0.81) mm/m in the SAVR group vs 1.11 (0.44, 1.78) mm/m in the TAVI group, *P* = 0.001 (Table 3 and Figs 1–3). Univariable and multivariable linear mixed model analyses revealed that TAVI procedure, baseline aortic diameter and paravalvular leak were significant risk factors for progression of AA dilatation after AVR (Table 4).

**Table 3:** Aortic diameters

SAVR				TAVI			
	Baseline	Follow-up	Diff-1 (95 CL)	Baseline	Follow-up	Diff-2 (95 CI)	*P*-value*
AA (mm)	39 (35, 42)	39.9 (36, 43)	0.95 (0.60, 1.30)	40 (37, 43.8)	41.6 (37, 45)	1.65 (0.67, 2.63)	0.02
AA/BSA (mm/m2)	20.9 (19, 22.7)	21.6 (19, 23)	0.72 (0.38, 1.05)	21.8 (20.6, 24)	22.8 (21, 25)	1.05 (0.39, 1, 71)	0.02
AA/h (mm/m)	22.7 (20.8, 24.6)	23.2 (21, 25)	0.59 (0.36, 0.81)	23.9 (22.8, 26.8)	25 (23, 27)	1.11 (0.44, 1.78)	0.001

* *P*-value between Diff-1 (SAVR) and Diff-2 (TAVR).

AA: ascending aorta; AA/BSA: ascending aorta diameter indexed to body surface area; AA/h: ascending aorta diameter indexed to height; Diff: difference; SAVR: surgical aortic valve replacement; TAVI: transcatheter aortic valve implantation.CI:Confidence Interval

**Table 4:** Ascending aorta diameter changes after aortic valve replacement using univariable and multivariable mixed model

	Univariable	Multivariable
	Beta (SE), *P*-value	Beta (SE), *P*-value
Age	0.008 (0.01), 0.409	
Female gender	0.31 (0.21), 0.138	
Hypertension	1.71 (1.21), 0.167	
Severe aortic regurgitation	0.06 (0.33), 0.836	
Baseline MPG (mmHg)	0007 (0.006), 0.304	
Baseline AA diameter (mm)	0.98 (0.21), 0.001	1.04 (0.05), <0.001
TAVI	0.71 (0.31), 0.002	1.45 (1.25), 0.024
Paravalvular leak	0.81 (0.33), 0.026	1.67 (0.67), 0.013

AA: ascending aorta; MPG: mean pressure gradient; SE: standard error; TAVI: transcatheter aortic valve implantation.

## DISCUSSION

BAV calcifies more frequently and earlier than tricuspid AV, consequently patients with BAV present an increased risk of AV degeneration and stenosis with possible indication to AVR [4–8]. Besides the increased risk of AV dysfunction, BAV is associated with a higher growth rate of AA diameter with an increased risk of AA aneurysm and dissection. This risk further increases with concomitant AV stenosis [4–8, 12–14].

The pathophysiology of AA dilatation in patients with BAV is still unclear, being related both to genetic and haemodynamic factors. The aorta of BAV patients typically presents a congenital wall weakness because of the reduced expression of fibrillin-1 and increased presence of matrix metalloproteinases associated with smooth muscle cell detachment and cellular apoptosis. Patients often present mutations in the NOTCH 1 gene (chromosome 9q) leading to abnormalities that may be responsible of the development of the bicuspid valve morphology and of the accelerated calcium deposition. Mutations of the ACTA 2 gene (chromosome 10q), which encodes the vascular smooth muscle cells alfa-actin, are associated with familial thoracic aneurysms and BAV. Consequent to histologic changes is the development of cystic medial necrosis, non-inflammatory loss of vascular smooth muscle cells of the medial aortic wall layer, fragmentation of the elastic fibres with depletion and disjunction of vascular smooth cells [4, 5, 7, 8]. Furthermore the aortic asymmetric wall stress caused by the BAV, together with the turbulent flow caused by the asymmetric valve, may favour dilatation of the AA. The turbulence of transaortic flow is further enhanced in patients with AV stenosis, by creating a high-velocity jet that increases shear stress on the antero-lateral portion of the AA, favouring aortic aneurysm development [4–8, 12–17].

BAV and the resulting aortopathy are characterized by a complex and varied pathophysiological mechanism. Studies performed using 4D flow Magnetic Resonance Imaging showed that different BAV phenotypes (such as fusion between the right and left cusp, R-L or between the right and non-coronary artery, R-NC) are associated with different postvalvular flow patterns. In other words, the direction of postvalvular blood jet seems to be influenced by the position of the fused cusp, with a different wall shear stress on the aortic wall and a consequent different evolution of the AA dilation. Therefore, the aortic wall/jet impingement locations correspond to regions of high wall shear stress, and the areas of the aortic wall with high shear stress are those most affected by dilation [15, 24]. For the same reason, different prostheses with various designs and haemodynamic characteristics could generate different flow patterns, with different location of wall shear stress on the aortic wall, causing dilations of the AA of different morphology. Further studies would confirm this hypothesis.

The prevalence of AA dilatation in patients with BAV ranges from 20% to 84% (mean value 50%), with an aortic growth rate of 0.39–0.77 mm/year, which is 2–4 times faster than in patients with tricuspid AV [4, 5, 12–19, 25]. Presently, SAVR is the gold standard for younger and operable patients with severe BAV stenosis. However recently in patients with BAV, TAVI has been proposed and favourable results have been reported [8, 10, 11]. In this study, in line with other Authors, we found a higher incidence of paravalvular leak and pacemaker implantation in the TAVI group [26, 27]. SAVR also presents a significantly better long-term survival and improvement of clinical status; however, the selection criteria including older age and worse baseline clinical conditions of patients of the TAVI group should be considered a limitation in the 2 groups comparison.

AA diameter progression after AVR is still controversial. Limited studies failed to show a significant progression of AA diameters in patients who underwent tricuspid or bicuspid AVR (SAVR or TAVI); therefore transvalvular haemodynamics more than genetic characteristics of the aortic wall seem to influence the development of AA dilatation [10, 11, 13, 14]. According to the Authors the most important contributing factor to the development of aortic dilatation in BAV patients is the haemodynamic wall stress subsequent to pathological transvalvular flow characteristics. By contrast, Russo *et al.* [18] and Yasuda *et al.* [14] reported in BAV patients a progressive dilatation of the AA after AVR, and it was significantly higher than tricuspid AV patients. In their opinion in BAV patients, AVR does not prevent progressive aortic dilatation, that persist because of the BAV-related aortopathy. A recent retrospective study of Hiraoka *et al.* [1] reported a significatively greater enlargement of the AA after AVR in BAV patients compared to tricuspid AV patients (0.46 vs 0.09 mm/year, *P* ≤ 0.001). As BAV dysfunction, such as AV stenosis, tends to reveal earlier than tricuspid ones, when patients are referred to surgery for AVR, AA diameter is often below the surgical indication and AA does not deserve surgical treatment. There is still a lack of data regarding the AA diameter progression or the risk of aortic events in BAV patients with normal-sized or moderately-dilated AA at the time of AVR. And, so far, it has never been investigated and there is no information regarding possible differences in the rate of aneurysmal progression in patients with BAV undergoing SAVR versus TAVI. In this study a significant increase of aortic diameter after AVR was observed in both groups. Given the higher aortic dilatation rate in BAV patients, this suggests a relevant influence of congenital weakness of the aortic wall on progressive aortic dilatation. Patients who underwent SAVR showed significantly less AA diameter progression, when compared to patients who underwent TAVI. Such difference was confirmed for AA diameter, AA/h and AA/BSA.

As expected, the results should be interpreted with caution. The different preoperative characteristics of the 2 groups of patients, such as age, initial aortic diameters and different comorbidities, may represent a limitation in obtaining definitive results. However, the aim of the study is to represent the ‘real-world’ scenario, in which TAVI patients are usually older and in worse clinical conditions than patients referred to SAVR. Furthermore, most of the patients were enrolled before the latest 2021 ESC/EACTS Guidelines for the Management of Valvular Heart Disease, where, differently from the previous Guidelines, the age threshold of patients referred to TAVI, after Heart Team evaluation, was 75 years (class of recommendation I, level of evidence A) [9].

Supposedly in patients undergoing TAVI, preservation of the native calcified cusps with possible imperfect adherence of the prosthesis to the bicuspid irregular annulus, and possible non-spherical expansion of TAVI prosthesis in BAV annuli, could favour a more turbulent and asymmetrical transprosthetic flow, responsible for greater wall stress on the congenitally weakened aortic wall [28]. Furthermore, supposedly, balloon-expandable valves may have more downstream turbulence due to their increased transvalvular gradients compared to self-expanding valves [29], dedicated studies are required. It may also be worth considering that percutaneous aortic prostheses, in particular self-expandable ones, after deployment may exert a constant radial force on the initial tract of the AA wall, which could contribute to its progressive dilatation. Further investigation would be required to clarify these notions.

A recent study of He *et al.* [8] comparing AA dilatation rate after TAVI in BAV versus tricuspid AV patients, at multivariate linear regression analysis, reported that paravalvular leak was the only independent predicting factor associated with AA dilatation rate in BAV patients (coefficient = 0.247, standard error = 0.083, *P* = 0.003). The study suggested that paravalvular regurgitation may be responsible for altered haemodynamics, thus increasing the asymmetric wall stress and subsequent AA dilatation. As previously reported [26–28], and confirmed in this study, TAVI patients present a significantly increased risk of paravalvular regurgitation when compared to SAVR, and consequently a higher risk of additional turbulent flow contributing to the increased AA dilatation in patients with bicuspid aortopathy. It is also conceivable that in SAVR patients the running suture for AA closure after prosthesis implantation, and the subsequent surgical scar, may play a containing role on the aortic wall, thus limiting its late dilatation. Indeed, in surgical patients, the larger AA diameter was observed in the middle tract of AA, quite far (1 and 2 cm) from the aortic running suture. Furthermore, due to differences in terms of haemodynamics, a possible difference between mechanical and biological surgical prostheses in terms of late dilation of the AA could be considered plausible. However, future dedicated studies with appropriate investigations could confirm these hypotheses.

### Limitations

This study has some limitations. The research is a non-randomized study, with a relatively limited number of patients. Based on the non-randomized nature of the study, baseline conditions of SAVR and TAVI patients were different, with TAVI patients being older, in worse clinical conditions and having a slightly higher preoperative AA diameter.

Furthermore, the diameters were analysed with transthoracic echocardiogram. Although the echocardiographic examinations were performed by a core-lab of expert cardiologists, and the echocardiogram, as previously reported, is considered a reliable tool for estimating aortic diameters [21, 22], the gold standard remains the angio CT scan, and this could represent a further limitation. Furthermore, beside BSA and height, also aortic length (centreline, from annulus to innominate artery takeoff), has been proposed as a predictor of acute aortic events, however this study was conceived before the 2022 ACC/AHA Guideline for the Diagnosis and Management of Aortic Disease [30]. However, this is the first study comparing AA diameter late progression after AVR (SAVR versus TAVI) for BAV stenosis, providing new and possibly useful information. Due to the trend of extending indication of TAVI to younger patients, more multicentre comparative studies with patients having more similar preoperative characteristics seem indicated and opportune.

## CONCLUSIONS

Compared to TAVI patients, BAV patients undergoing SAVR show less AA dilatation after AVR. The lower tendency to late AA dilatation seems to confirm that, in operable patients with good life expectative, presenting BAV stenosis and indication to AV treatment, SAVR should be preferred to TAVI to reduce the risk of increased late AA dilatation.

## Supplementary Material

ivae100_Supplementary_Data

## Data Availability

The data underlying this article are available in the article and in its Supplementary Material.

## Notes

Presented at the 37th EACTS Annual Meeting, October 4-7, 2023, Vienna, Austria.

## ABBREVIATIONS

AA: Ascending aorta
AA/BSA: Ascending aorta diameter indexed to body surface area
AA/h: Ascending aorta diameter indexed to height
AV: Aortic valve
AVR: Aortic valve replacement
BAV: Bicuspid aortic valve
BSA: Body surface area
EOA: Effective orifice area
NYHA: New York Heart Association
SAVR: Surgical aortic valve replacement
TAVI: Transcatheter aortic valve implantation
