# Supplementary material for: Progression of the ascending aorta diameter after surgical or transcatheter bicuspid aortic valve replacement
Source: Interdiscip Cardiovasc Thorac Surg. 2024 May 22;38(5):ivae100. doi: 10.1093/icvts/ivae100 (PMC11142625; doi:10.1093/icvts/ivae100)
Supplement: ivae100_Supplementary_Data [file ivae100_supplementary_data.zip › ivae100_Supplementary_Data.docx]

|  |
| --- |

Table S1. Operative details

| Characteristic | TAVI, N = 46 | SAVR, N = 143 |
| --- | --- | --- |
| Type of prosthesis |  |  |
| TAVR n(%) |  |  |
| *Self-expandable* | 36 (78) |  |
| Corevalve Evolut R | 15 (33) |  |
| Corevalve Evolut Pro | 21 (45) |  |
| *Balloon-expandable* | 10 (22) |  |
| Edwards Sapien 3 | 8 (17) |  |
| Edwards Sapien 3 Ultra | 2 (5) |  |
| SAVR n(%) |  |  |
| *Bioprosthesis* |  | 119 (93) |
| Avalus |  | 4 (3) |
| Biointegral |  | 4 (3) |
| Epic |  | 8 (6) |
| Inspiris Resilia |  | 7 (5) |
| Magna Ease |  | 61 (43) |
| Trifecta |  | 35 (24) |
| *Sutureless-Rapid Deployment Bioprosthesis* |  | 2 (1.4) |
| INTUITY Elite |  | 1 (1) |
| Perceval |  | 1 (1) |
| *Mechanical* |  | 18 (13) |
| Regent |  | 14 (10) |
| Carbomedics |  | 8 (6) |
