# Supplementary figures and images for: Progression of the ascending aorta diameter after surgical or transcatheter bicuspid aortic valve replacement
Source: Interdiscip Cardiovasc Thorac Surg. 2024 May 22;38(5):ivae100. doi: 10.1093/icvts/ivae100 (PMC11142625; doi:10.1093/icvts/ivae100)

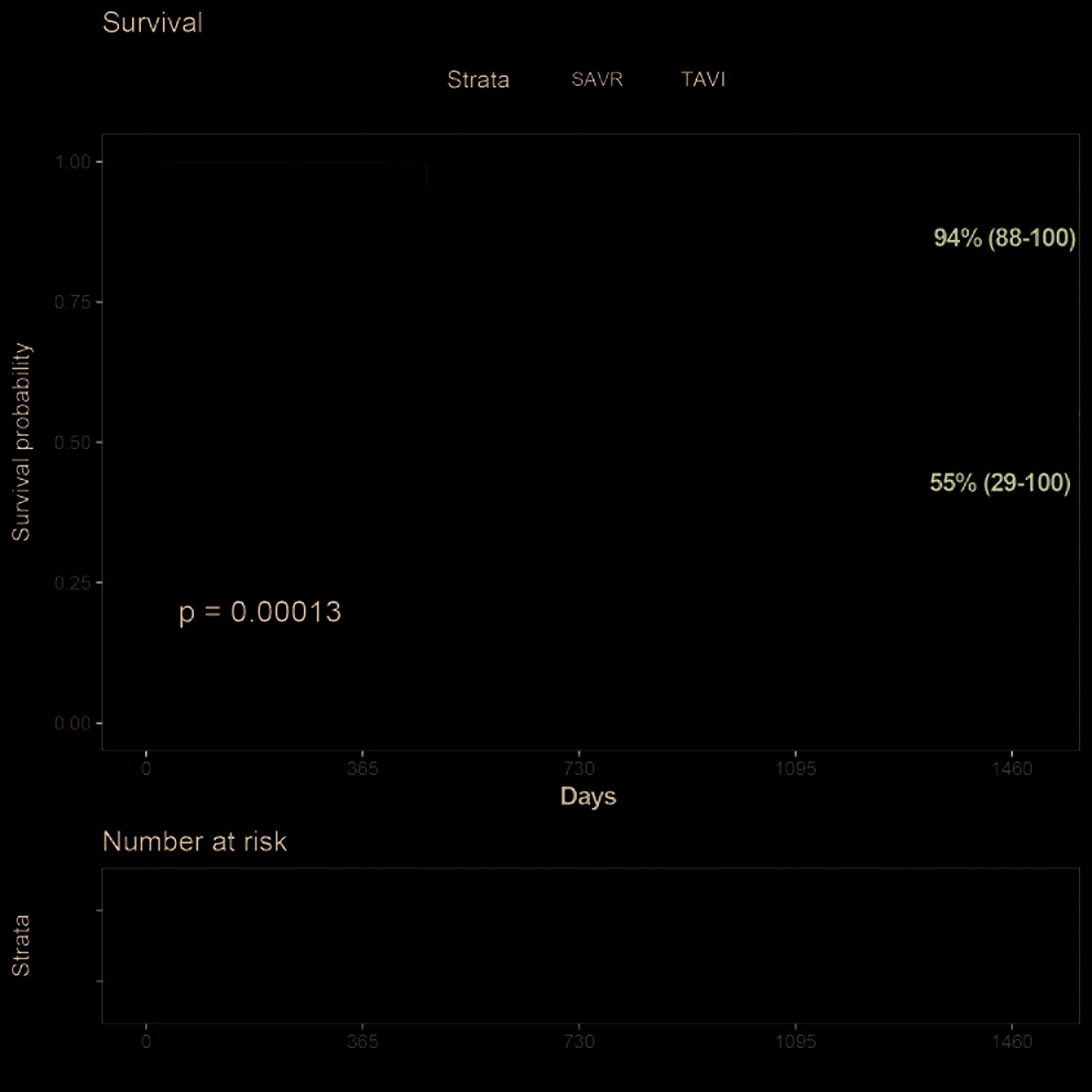

Supplement: ivae100_Supplementary_Data [file ivae100_supplementary_data.zip › FIGURE SR-1.tif]

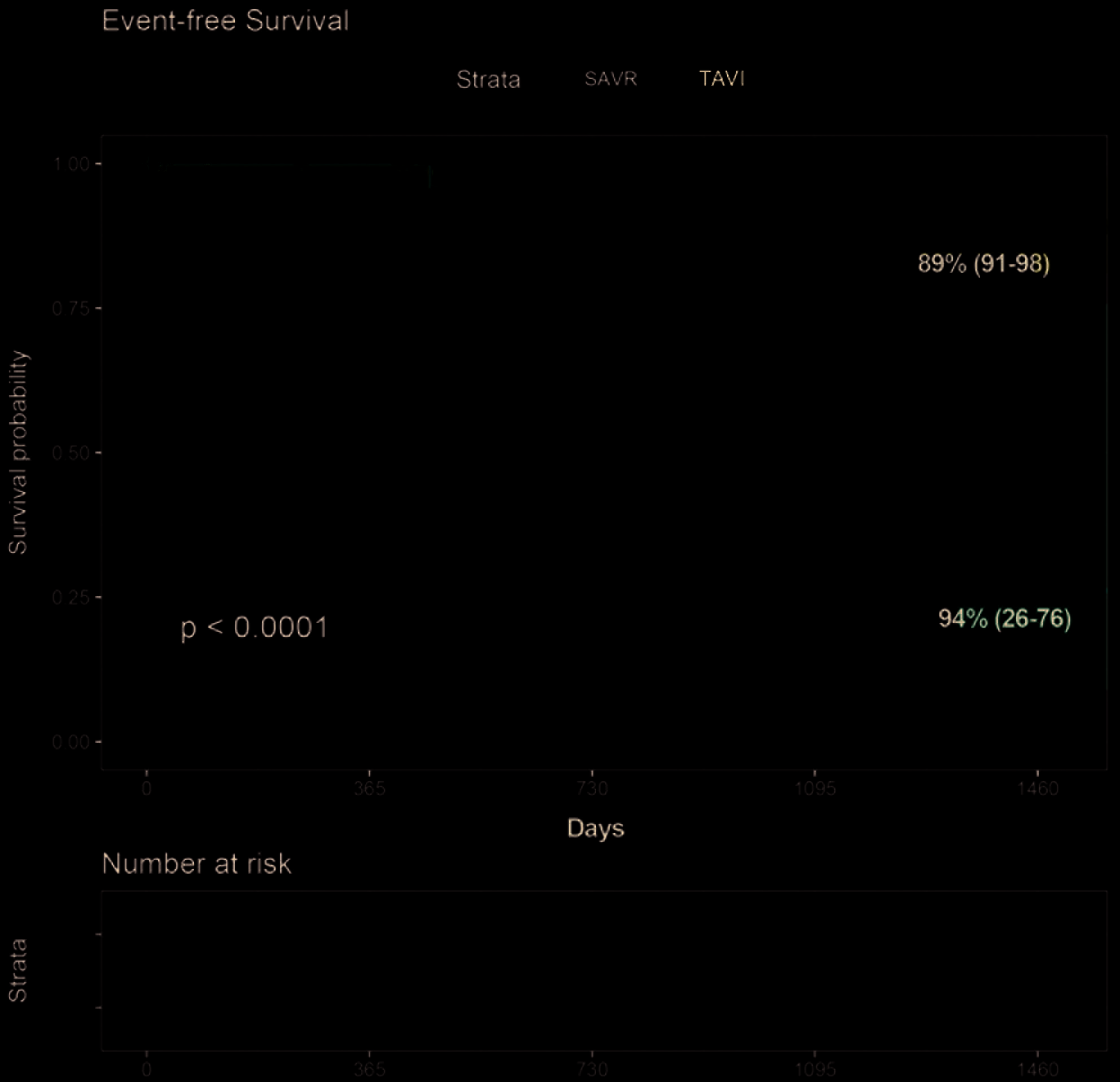

Supplement: ivae100_Supplementary_Data [file ivae100_supplementary_data.zip › FIGURE SR-2.tif]
